# Activation of AMPK/mTOR-Driven Autophagy and Suppression of the HMGB1/TLR4 Pathway with Pentoxifylline Attenuates Doxorubicin-Induced Hepatic Injury in Rats

**DOI:** 10.3390/ph17060681

**Published:** 2024-05-26

**Authors:** Hany H. Arab, Ahmed H. Eid, Shuruq E. Alsufyani, Ahmed M. Ashour, Alwaleed M. Alnefaie, Nasser M. Alsharif, Abdullah M. Alshehri, Abdulmajeed A. Almalawi, Abdulmajeed A. Alsowat, Hayat A. Abd El Aal, Eman S. G. Hassan, Wessam H. Elesawy, Alzahraa A. Elhemiely

**Affiliations:** 1 Department of Pharmacology and Toxicology, College of Pharmacy, Taif University, P.O. Box 11099, Taif 21944, Saudi Arabia; 2 Department of Pharmacology, Egyptian Drug Authority (EDA)—Formerly NODCAR, Giza 12654, Egypt; 3 Department of Pharmacology and Toxicology, College of Pharmacy, Umm Al Qura University, P.O. Box 13578, Makkah 21955, Saudi Arabia; 4 College of Pharmacy, Taif University, P.O. Box 11099, Taif 21944, Saudi Arabia; 5 Department of Pharmacology and Toxicology, College of Pharmaceutical Sciences and Drug Manufacturing, Misr University for Science and Technology, Giza 12568, Egypt

**Keywords:** pentoxifylline, doxorubicin, hepatotoxicity, autophagy, AMPK, HMGB1

## Abstract

Despite being an effective chemotherapeutic agent, the clinical use of doxorubicin (DOX) is limited by several organ toxicities including hepatic injury. Pentoxifylline (PTX) is a methylxanthine derivative with marked anti-inflammatory and anti-apoptotic features. It is unknown, however, whether PTX can mitigate DOX-evoked hepatotoxicity. This study aims to explore the potential hepatoprotective impact of PTX in DOX-induced hepatic injury and the underlying molecular mechanisms. Histopathology, immunohistochemistry, and ELISA were used to examine liver tissues. The current findings revealed that PTX administration to DOX-intoxicated rats mitigated the pathological manifestations of hepatic injury, reduced microscopical damage scores, and improved serum ALT and AST markers, revealing restored hepatic cellular integrity. These favorable effects were attributed to PTX’s ability to mitigate inflammation by reducing hepatic IL-1β and TNF-α levels and suppressing the pro-inflammatory HMGB1/TLR4/NF-κB axis. Moreover, PTX curtailed the hepatic apoptotic abnormalities by suppressing caspase 3 activity and lowering the Bax/Bcl-2 ratio. In tandem, PTX improved the defective autophagy events by lowering hepatic SQSTM-1/p62 accumulation and enhancing the AMPK/mTOR pathway, favoring autophagy and hepatic cell preservation. Together, for the first time, our findings demonstrate the ameliorative effect of PTX against DOX-evoked hepatotoxicity by dampening the hepatic HMGB1/TLR4/NF-κB pro-inflammatory axis and augmenting hepatic AMPK/mTOR-driven autophagy. Thus, PTX could be utilized as an adjunct agent with DOX regimens to mitigate DOX-induced hepatic injury.

## 1. Introduction

Doxorubicin (DOX), also known as adriamycin, is an effective chemotherapeutic agent that belongs to the anthracycline antibiotic family. It is used in the clinical setting for a number of solid and hematological malignancies, including lymphoma and leukemia, alongside breast, lung, thyroid, and colon cancers [1]. As with most anticancer agents, DOX has a high incidence of serious adverse effects. In this context, there are dose-limiting cardiotoxic manifestations and severe myelosuppression that warrant a boxed warning on its packaging [2]. DOX, on the other hand, adversely affects other organs of the body such as the liver; a principal organ for drug detoxification [3]. Hence, the quest to understand the molecular basis for DOX-mediated toxicities and to find effective treatments continues to be a challenge for scientists [4].

The liver, which is the body’s main metabolic organ and the first line of defense for drug detoxification, is among the first organs affected by DOX therapy [5]. Ample evidence exists that DOX induces hepatotoxicity in cancer patients alongside experimental animals [1,3,4,5,6]. During its metabolism in the liver, DOX undergoes extensive transformations, with doxorubicinol being the most toxic metabolite [2,3]. Notably, DOX-induced toxicity is a multifactorial phenomenon that has not yet been fully characterized. It is still believed that such toxicity is initiated by oxidative stress, which is associated with excessive generation of reactive oxygen species (ROS) and mitochondrial dysfunction [7]. Moreover, the instigation of inflammatory cascades by DOX is a contributing factor to hepatic toxicity, as well as programmed cellular death (apoptosis) [1,5]. In this regard, hepatic inflammation evoked by DOX treatment is well characterized by sustained activation and upregulation of the nuclear factor kappa B (NF-κB). This transcription factor controls the generation of interleukin-1 beta (IL-1β) and tumor necrosis-α (TNF-α) pro-inflammatory cytokines, which further intensify hepatic inflammation [1,3,4,6]. In the context of inflammation, the necrotic hepatocytes and macrophages release several damage-associated molecular patterns (DAMPs), including the high-mobility group box 1 (HMGB1) that switches on innate immunity. By binding to the toll-like receptors (TLRs), HMGB1 stimulates NF-κB signaling. As a result, excessive levels of proinflammatory chemokines and cytokines are generated, culminating in an intensified inflammatory response [8]. Notably, it has not been previously investigated whether HMGB1/TLR4 may be involved in the pathogenesis of DOX-evoked hepatic injury. With respect to apoptotic cell death, DOX has been reported to trigger apoptosis by activating the intrinsic (mitochondrial) pathway with the depletion of anti-apoptotic B-cell lymphoma 2 (Bcl-2) family signals and thus enhancing mitochondrial membrane permeabilization, culminating in caspase 3 activation and cell death [3,4,5,6]. 

In response to intracellular and extracellular stressors, macroautophagy (referred to as autophagy hereafter) maintains cellular homeostasis by prompting cell survival. In perspective, the “autophagy flux” can be described as a self-degrading process that involves the sequestration of damaged organelles/proteins into double-membraned autophagosomes that fuse with lysosomes for degradation by resident hydrolases [9]. In the context of DOX-induced organ toxicity, contradictory findings have emerged regarding autophagy events where both autophagy stimulation [10,11] and inhibition [1,12,13] have been described. In rodents, autophagy dysregulation has been implicated in mediating tissue damage [1]. For instance, impeded autophagy has been characterized as a major player in interceding DOX-induced cardiotoxicity. In this regard, the blockade of autophagy flux by DOX has been reported at the lysosome level, preventing lysosomes from processing proteotoxic loads despite the initial stimulation of autophagy responses [1,12,13]. Likewise, the upstream regulatory signals responsible for driving the autophagy events, such as the mechanistic target of rapamycin (mTOR) and AMP-activated protein kinase (AMPK), are deregulated by DOX, resulting in marked toxicity [1,9]. On the other hand, the literature revealed that autophagy stimulation can mediate DOX-induced toxicity [10,11]. Hence, further research is needed to determine how autophagy contributes to the toxicity associated with DOX. More importantly, it is unclear whether autophagy plays a crucial role in DOX-induced hepatic injury in vivo. Thus, this study aimed to examine the potential effect of doxorubicin on hepatic autophagy in vivo in rats.

Several studies have reported that autophagy and apoptosis are tightly regulated in order to maintain tissue homeostasis [14,15]. During autophagy, organelles and proteins are degraded by lysosomes in order to ensure cell survival in the presence of several cellular stressors [9], whereas, during apoptosis, damaged or mutant cells are eliminated [1,15]. Hence, cellular fate is determined by the crosstalk between autophagy and apoptosis. In this regard, autophagy is believed to inhibit apoptosis in several inflammatory conditions, including liver injury [15]. In diabetic rats with liver dysfunction, the pro-inflammatory HMGB1 is one of several signals that modulate the interplay between apoptosis and autophagy [9,15]. Indeed, HMGB1 regulates autophagosome formation by binding to the autophagy signal Beclin1, thereby serving as an autophagy effector [16].

**Figure 1:**
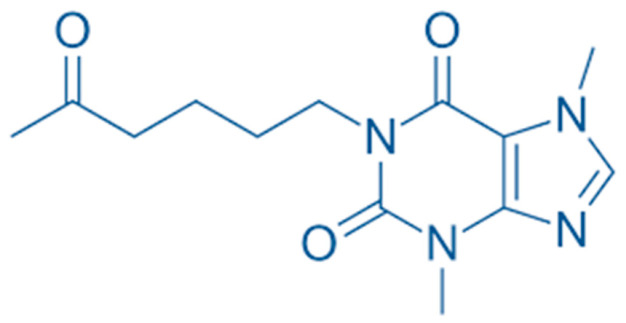
Chemical structure of pentoxifylline.

Pentoxifylline (PTX) is a methylxanthine derivative that serves as a nonspecific phosphodiesterase inhibitor (the chemical structure is illustrated in Figure 1). Due to its high safety/tolerability and the lack of a serious drug–drug interaction, PTX has been applied in clinical settings since 1972 for the management of vascular diseases, including peripheral vascular disease, defective regional microcirculation, and cerebrovascular disease [17,18]. There is, however, a possibility that PTX can reduce plasma fibrinogen levels, an event that may lead to bleeding in patients with platelet dysfunction [19]. Beyond the classical use of PTX, it elicits beneficial effects on the course of various experimental inflammatory disorders due to its anti-inflammatory and anti-apoptotic properties [18,20,21]. In rodent models, PTX has been reported to dampen the pathological signs associated with lung injury caused by chlorine [18] or cross-clamping of the infrarenal aortic artery [21] as well as acute kidney injury caused by rhabdomyolysis [19]. In the context of hepatic injury, several studies have indicated that PTX elicits a beneficial effect against D-galactosamine-induced hepatotoxicity [22] and non-alcoholic steatohepatitis (NASH) [23], while other reports revealed that PTX does not prevent or even exacerbate NASH [21,24]. These experimental data coincide with the clinical data that revealed that PTX mitigates NASH, while other studies have reported that PTX does not prevent or even worsen hepatic injury in some patients [25]. In addition to this unresolved controversy regarding PTX’s possible effects against hepatic injury, no previous studies have been conducted on the efficacy of PTX against DOX-induced hepatic injury. In an attempt to bridge this gap, this study aims to explore the potential hepatoprotective impact of PTX in DOX-induced hepatic injury and the molecular mechanisms pertaining to AMPK/mTOR-driven autophagy and HMGB1/TLR4/NF-κB-associated inflammation. To this end, hepatic injury was examined by histopathology and serum liver function tests. At the molecular levels, the inflammatory response was evaluated by measuring pro-inflammatory cytokines and exploring the HMGB1/TLR4/NF-κB axis. Moreover, autophagy events were examined by quantifying Beclin 1 and SQSTM-1/p62 and exploring the AMPK/mTOR pathway. In tandem, hepatic apoptotic changes were investigated by determining caspase 3 activity and B-cell lymphoma 2-associated X protein (Bax)/Bcl-2 ratio. Overall, the current findings revealed that PTX mitigated hepatic injury by suppressing the HMGB1/TLR4/NF-κB pro-inflammatory axis and augmenting hepatic AMPK/mTOR-driven autophagy. 

## 2. Results

### 2.1. PTX Improved Hepatic Pathological Signs and Hepatic Cellular Integrity Markers in DOX-Intoxicated Rats

**Figure 2:**
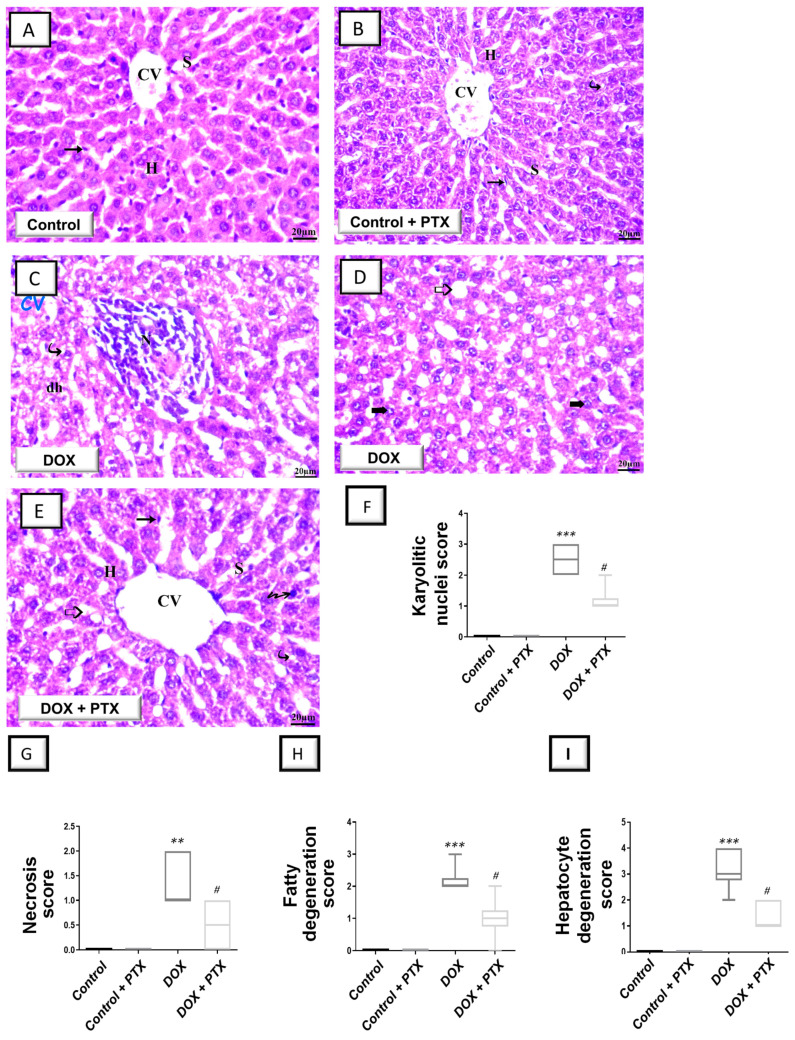
Pentoxifylline mitigated hepatic histopathological changes in doxorubicin-intoxicated rats. Pentoxifylline (PTX; 100 mg/kg) was orally received daily, while doxorubicin (DOX; 18 mg/kg total cumulative dose; 3 mg/kg dose weekly) was received by the intraperitoneal route. The treatments were administered for 6 weeks. As examined by H–E staining in the hepatic tissues of control rats (**A**) and PTX-treated rats (**B**), these tissues showed normal morphology with a distinctive central vein (CV), normal hepatocyte layout (H), and Kupffer cells (arrow). Within the cells, hepatocyte nuclei were seen as dark red structures, whilst light red staining was observed in the cytoplasm. Moreover, typical sinusoids (S) and regular portal region were present (images at 400× magnification; scale bar: 20 µm). (**C**,**D**) H–E staining in the hepatic tissue of DOX-intoxicated rats revealed marked histopathological alterations including hepatocyte degeneration (dh), necrosis (N), fatty degeneration (hollow arrow), and karyolitic nuclei (bold arrow; 400× magnification; scale bar: 20 µm). (**E**) H–E staining in the hepatic tissue of the PTX-treated DOX-intoxicated group demonstrated improved hepatic pathological signs and hepatic tissue preservation. However, fatty degeneration (hollow arrow), pyknotic cells (zigzag arrow), and cytoplasmic vacuoles (curved arrow) were still observed in some focal areas (400× magnification; scale bar: 20 µm). (**F**–**I**) Histopathological damage scores, including scores of karyolitic nuclei, necrosis, fatty degeneration, and hepatocyte degeneration. Statistical analysis of the results (expressed as the median with interquartile range; *n* = 6) was conducted by Kruskal–Wallis analysis and Dunn’s test. ** *p* < 0.01, or *** *p* < 0.001 vs. control; ^#^ *p* < 0.05 vs. DOX group. PTX, pentoxifylline; DOX, doxorubicin.

Initially, hematoxylin and eosin (H–E) staining was conducted to observe gross abnormalities in the liver tissue of DOX-intoxicated animals under a light microscope. As illustrated in Figure 2A,B, sections from the control group and PTX-treated control group revealed a normal structure with no histopathological alterations and a distinctive central vein and hepatocyte layout. Within cells, hepatocyte nuclei were seen as dark red structures, whilst light red staining was observed in the cytoplasm. Moreover, sinusoids and the regular portal region were present. As depicted in Figure 2C, the hepatic tissue collected from DOX-intoxicated rats showed marked histopathological alterations including hydropic degraded cells, altered lobular form, nuclear deterioration in certain areas, moderate fatty degeneration, disarrangement of ordinary hepatic cells, necrosis, and inflammatory cell influx. In the DOX-intoxicated group, the hepatic central vein and portal vein were enlarged and congested and evident hepatic cell disintegration was observed (karyolitic nuclei). Together, these pathological signs reveal the successful establishment of DOX-induced hepatic injury. As illustrated in Figure 2E, the PTX-treated DOX-intoxicated group demonstrated improved hepatic histopathological signs with preservation of hepatic tissue. However, some focal areas demonstrated the presence of fatty degeneration, pyknotic cells, and cytoplasmic vacuoles. 

Several histopathological signs were quantified in animals as karyolitic nuclei, necrosis, fatty degeneration, and hepatocyte degeneration (Figure 2F–I) to characterize hepatic histopathological damage further. In this regard, DOX significantly elevated the scores of karyolitic nuclei (*p* < 0.001), necrosis (*p* < 0.01), fatty degeneration (*p* < 0.001), and hepatocyte degeneration (*p* < 0.001). Following PTX administration to DOX-intoxicated animals, a significant reduction was seen (*p* < 0.05) in these scores, pointing to PTX’s ability to dampen hepatocyte histopathological aberrations. 

**Figure 3:**
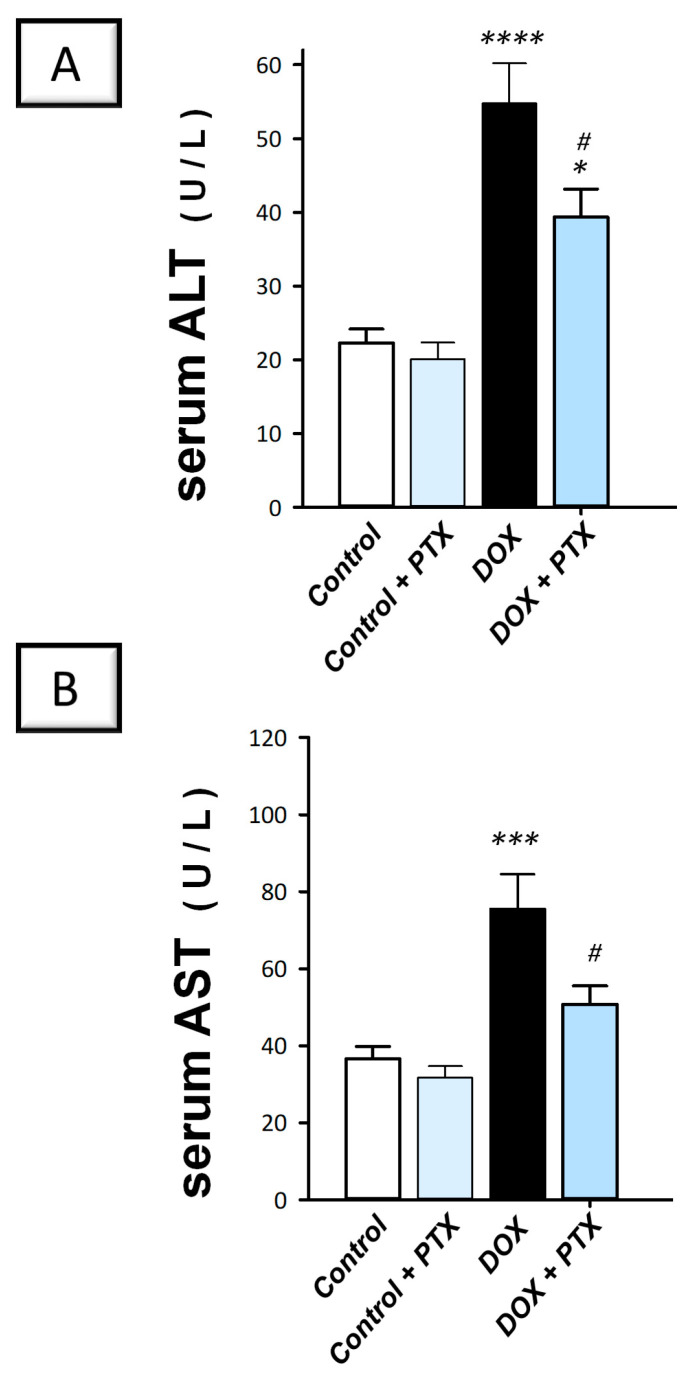
Pentoxifylline improved hepatic cellular integrity in doxorubicin-intoxicated rats. Pentoxifylline (PTX; 100 mg/kg) was orally received daily, while doxorubicin (DOX; 18 mg/kg total cumulative dose; 3 mg/kg dose weekly) was received by the intraperitoneal route. The treatments were administered for 6 weeks. Herein, serum ALT and AST were determined colorimetrically. In the hepatic tissue of DOX-intoxicated rats, PTX resulted in diminished serum ALT (**A**) and AST (**B**) activities, revealing an improved integrity of the hepatocytes. For *n* = 6, the values were shown as mean ± S.E.M. * *p* < 0.05, *** *p* < 0.001, or **** *p* < 0.0001 vs. control; ^#^ *p* < 0.05 vs. DOX group. PTX, pentoxifylline; DOX, doxorubicin.

In line with the histopathology findings, the DOX group deteriorated the liver cell integrity, as indicated by the significant elevation in serum alanine aminotransferase (ALT; *p* < 0.001) and aspartate aminotransferase (AST; *p* < 0.001) by 146% and 106.6%, respectively (Figure 3A,B). Following PTX administration to DOX-intoxicated animals, the raised serum activities of ALT and AST were significantly lowered (*p* < 0.05) by 28.1% and 32.8%, respectively. For ALT, these values were still significantly higher than the normal values of the control group. Together, these findings prove the competence of PTX to improve the liver pathological signs and hepatocyte-disrupted integrity of DOX-intoxicated rats. 

### 2.2. PTX Suppressed the Pro-Inflammatory Response in the Hepatic Tissue of DOX-Intoxicated Animals

**Figure 4:**
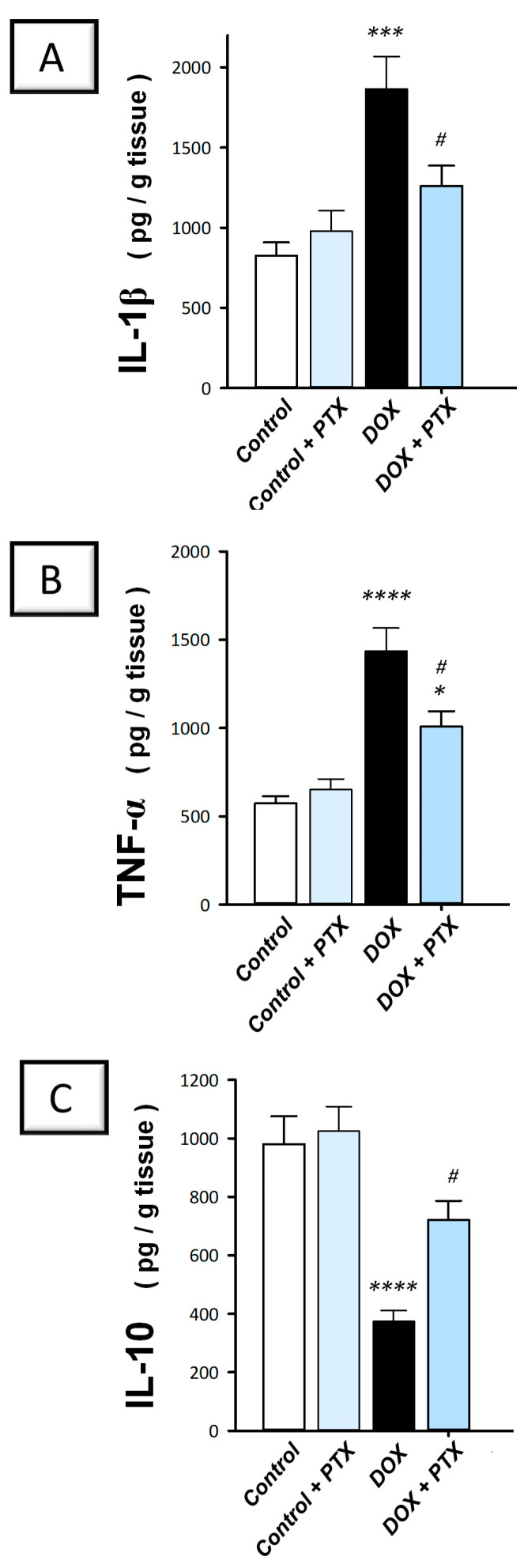
Pentoxifylline dampened the hepatic pro-inflammatory milieu in doxorubicin-intoxicated rats. Pentoxifylline (PTX; 100 mg/kg) was orally received daily, while doxorubicin (DOX; 18 mg/kg total cumulative dose; 3 mg/kg dose weekly) was received by the intraperitoneal route. The treatments were administered for 6 weeks. Herein, hepatic interleukin 1 beta (IL-1β), tumor necrosis factor-alpha (TNF-α), and interleukin 10 (IL-10) were quantified by ELISA. In the hepatic tissue of DOX-intoxicated rats, PTX resulted in diminished IL-1β (**A**) and TNF-α (**B**) levels and enhanced IL-10 (**C**). For *n* = 6, the values were shown as mean ± S.E.M. * *p* < 0.05, *** *p* < 0.001, or **** *p* < 0.0001 vs. control; ^#^ *p* < 0.05 vs. DOX group. PTX, pentoxifylline; DOX, doxorubicin.

The present findings revealed that DOX significantly upregulated the hepatic protein expression of IL-1β (*p* < 0.001) and TNF-α (*p* < 0.0001) by 126% and 150.8%, respectively (Figure 4A,B). In contrast, DOX significantly downregulated IL-10 protein levels (*p* < 0.0001) by 61.9% (Figure 4C), implying intensified pro-inflammatory events. Following PTX administration to DOX-intoxicated animals, the exaggerated inflammatory events were attenuated, as evidenced by the significant reduction (*p* < 0.05) in hepatic IL-1β and TNF-α by 32.4% and 29.8%, respectively, alongside the significant augmentation in IL-10 (*p* < 0.05) by 93.2%. These findings indicate that dampening the pro-inflammatory response is, at least partly, implicated in the favorable outcomes of PTX against DOX-evoked hepatotoxicity. 

### 2.3. PTX Inhibited the Inflammation-Linked HMGB1/TLR4/NF-κB Axis in the Hepatic Tissue of DOX-Intoxicated Rats

**Figure 5:**
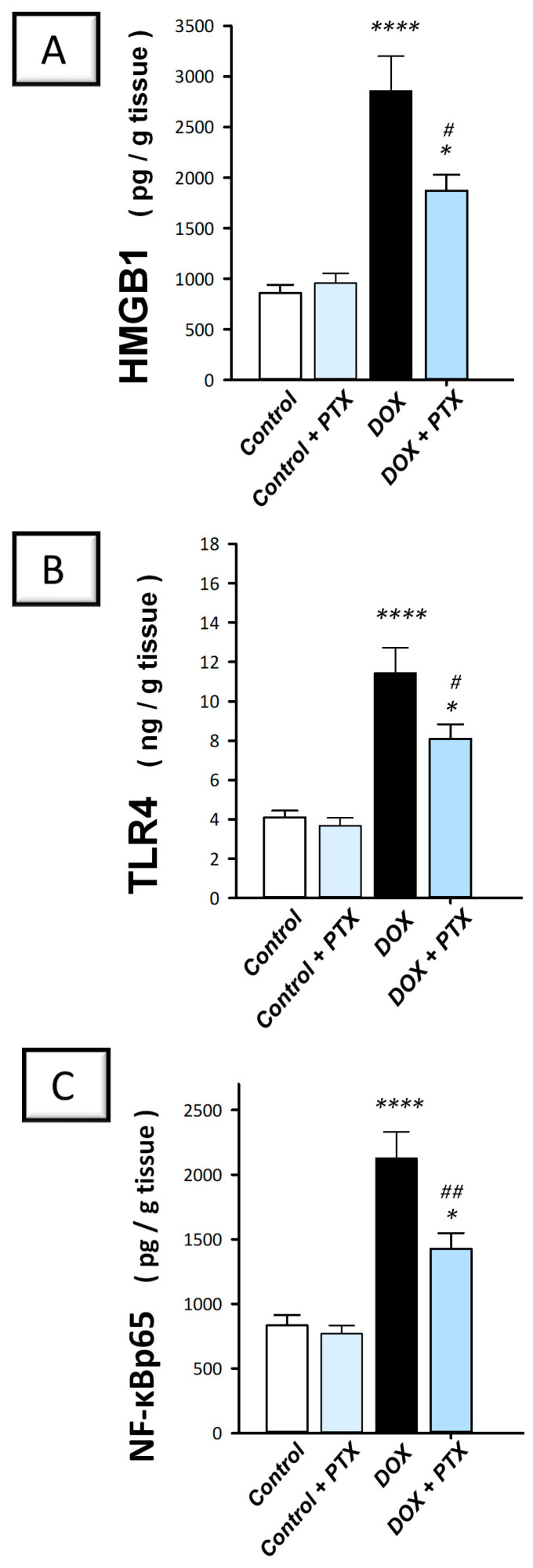
Pentoxifylline inhibited the HMGB1/TLR4/NF-κB axis in doxorubicin-intoxicated rats. Pentoxifylline (PTX; 100 mg/kg) was orally received daily, while doxorubicin (DOX; 18 mg/kg total cumulative dose; 3 mg/kg dose weekly) was received by the intraperitoneal route. The treatments were administered for 6 weeks. Herein, hepatic high mobility group box 1 (HMGB1), toll-like receptor 4 (TLR4), and nuclear factor kappa B- protein 65 (NF-κBp65) were quantified by ELISA. In the hepatic tissue of DOX-intoxicated rats, PTX downregulated the hepatic protein expression of HMGB1 protein (**A**), TLR4 (**B**), and NF-κBp65 (**C**), implying inhibition of the HMGB1/TLR4/NF-κB axis. For *n* = 6, the values were shown as mean ± S.E.M. * *p* < 0.05, or **** *p* < 0.0001 vs. control; ^#^ *p* < 0.05, or *^##^ p* < 0.01 vs. DOX group. PTX, pentoxifylline; DOX, doxorubicin.

It has been reported that the pro-inflammatory HMGB1 plays a crucial role in controlling autophagy [15]. Therefore, PTX was investigated for its effect on the expression of HMGB1 protein and its related TLR4/NF-B pathway. The present findings revealed that DOX significantly (*p* < 0.0001) elevated hepatic HMGB1, TLR4, and nuclear NF-κBp65 protein levels by 232.8%, 179%, and 155.3%, respectively (Figure 5A–C). Following PTX administration to DOX-intoxicated animals, the hepatic pro-inflammatory HMGB1/TLR4/NF-κB axis was inhibited, as evidenced by the significant reduction in HMGB1 (*p* < 0.05), TLR4 (*p* < 0.05), and the nuclear NF-κBp65 (*p* < 0.01) protein levels by 34.5%, 29.2%, and 32.8%, respectively. These findings reveal that dampening the pro-inflammatory HMGB1/TLR4/NF-κB axis is, at least partly, involved in its favorable outcomes against DOX-evoked hepatic toxicity. 

### 2.4. PTX Dampened the Pro-Apoptotic Machinery in the Hepatic Tissue of DOX-Intoxicated Rats

**Figure 6:**
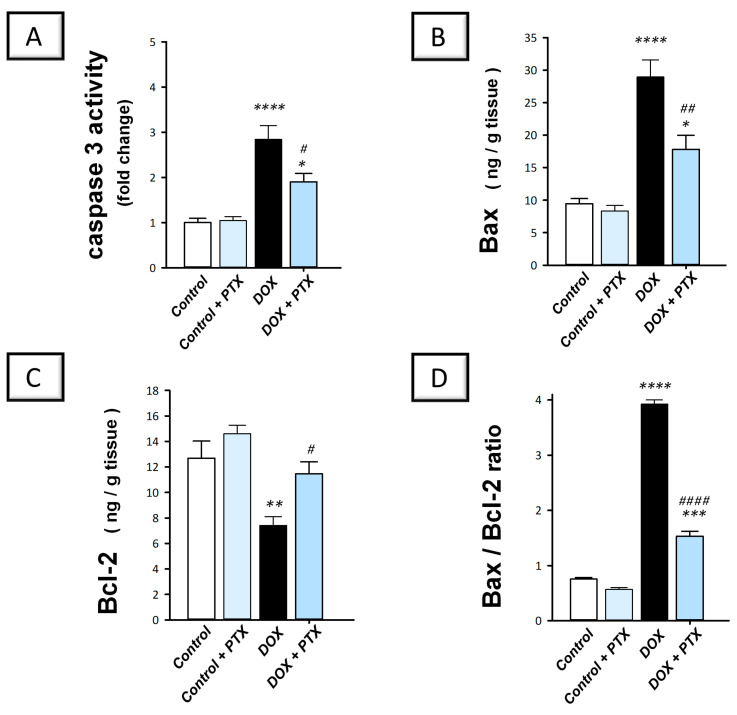
Pentoxifylline mitigated the hepatic pro-apoptotic events in doxorubicin-intoxicated rats. Pentoxifylline (PTX; 100 mg/kg) was orally received daily, while doxorubicin (DOX; 18 mg/kg total cumulative dose; 3 mg/kg dose weekly) was received by the intraperitoneal route. The treatments were administered for 6 weeks. Herein, caspase 3 activity was determined colorimetrically, while the content of Bcl-2 associated x (Bax) and B-cell lymphoma-2 (Bcl-2) was assayed by ELISA. In the hepatic tissue of DOX-intoxicated rats, PTX resulted in diminished caspase 3 activity (**A**) and Bax levels (**B**). Moreover, PTX enhanced Bcl-2 protein levels (**C**). Moreover, PTX lowered the Bax/Bcl-2 ratio (**D**). For *n* = 6, the values were shown as mean ± S.E.M. * *p* < 0.05, ** *p* < 0.01, *** *p* < 0.001, or **** *p* < 0.0001 vs. control; ^#^ *p* < 0.05, *^##^ p* < 0.01, or *^####^ p* < 0.0001 vs. DOX group. PTX, pentoxifylline; DOX, doxorubicin.

As reliable markers for apoptosis, we examined caspase 3 activity alongside Bax and Bcl2 protein expression to outline PTX’s impact on DOX-induced apoptosis. The present findings revealed that DOX significantly enhanced hepatic caspase 3 activity (*p* < 0.0001) by 184.2% (Figure 6A). Moreover, DOX significantly upregulated Bax protein expression (*p* < 0.0001) by 206.1% and significantly downregulated Bcl-2 protein expression (*p* < 0.01) by 41.6%, resulting in a spike of the Bax/Bcl-2 ratio (Figure 6B–D). These events imply intensified pro-apoptotic cell death response in hepatic tissue. Following PTX administration to DOX-intoxicated animals, the exaggerated apoptotic events were mitigated as evidenced by the significant increment in Bcl2 protein levels (*p* < 0.05) by 54.7%, alongside the significant reduction in caspase 3 activity (*p* < 0.05) and Bax protein expression (*p* < 0.01) by 33.1% and 38.5%, respectively. These data indicate that dampening the pro-apoptotic response by PTX is, at least partly, involved in its favorable outcomes against DOX-evoked hepatic toxicity. 

### 2.5. PTX Rescued the Impaired Autophagy Response in the Hepatic Tissue of DOX-Intoxicated Rats

**Figure 7:**
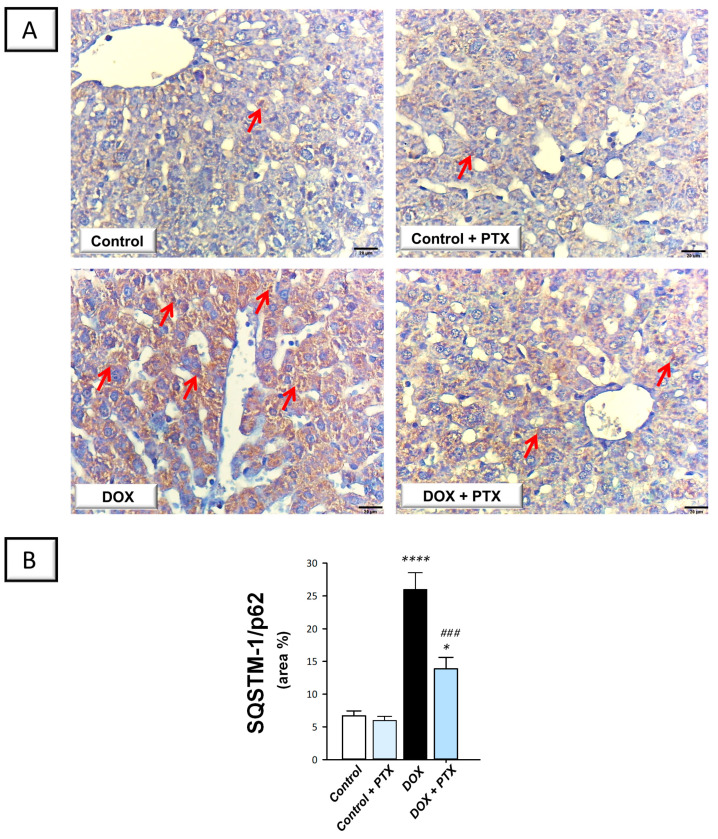
Pentoxifylline diminished hepatic accumulation of sequestosome-1/protein 62 (SQSTM-1/p62) in doxorubicin-intoxicated rats. Pentoxifylline (PTX; 100 mg/kg) was orally received daily, while doxorubicin (DOX; 18 mg/kg total cumulative dose; 3 mg/kg dose weekly) was received by the intraperitoneal route. The treatments were administered for 6 weeks. Herein, hepatic protein expression of SQSTM-1/p62 was determined by immunohistochemistry. (**A**) Immunohistochemistry micrographs of SQSTM-1/p62 protein expression in the hepatic tissue of animals (Red arrows indicate positive staining of target protein; images at 400× magnification; scale bar: 20 µm). (**B**) SQSTM-1/p62 protein quantification was shown in the bar chart as the area % (based on six non-overlapping microscopic fields). For *n* = 6, the values were shown as mean ± S.E.M. * *p* < 0.05, or **** *p* < 0.0001 vs. control; *^###^ p* < 0.001 vs. DOX group. PTX, pentoxifylline; DOX, doxorubicin.

**Figure 8:**
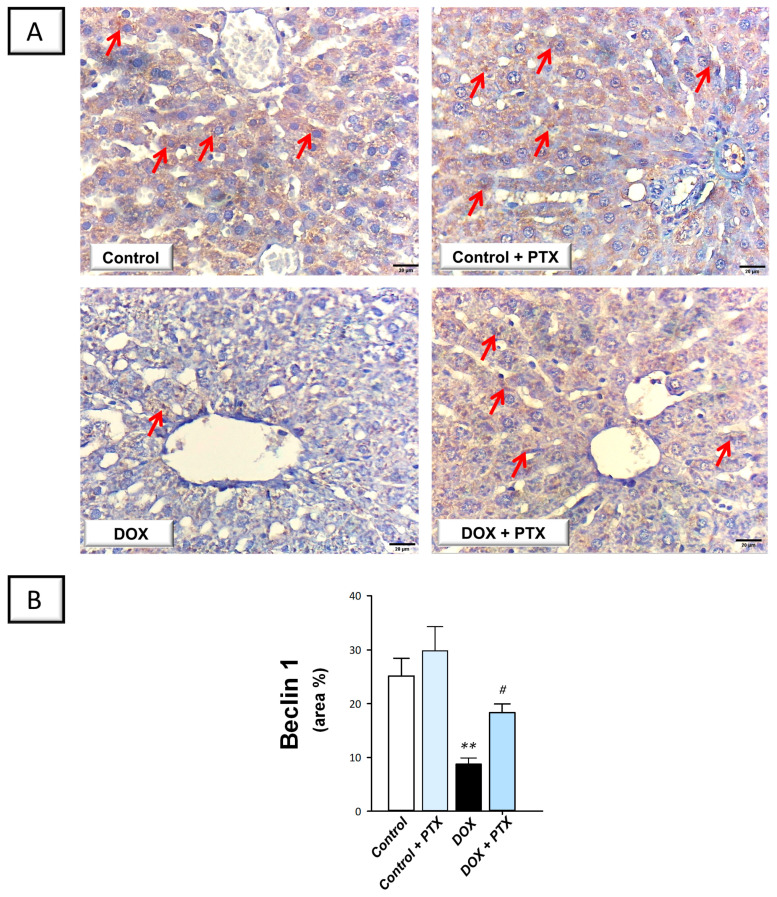
Pentoxifylline augmented hepatic Beclin 1 protein expression in doxorubicin-intoxicated rats. Pentoxifylline (PTX; 100 mg/kg) was orally received daily, while doxorubicin (DOX; 18 mg/kg total cumulative dose; 3 mg/kg dose weekly) was received by the intraperitoneal route. The treatments were administered for 6 weeks. Herein, hepatic protein expression of Beclin 1 was determined by immunohistochemistry. (**A**) Immunohistochemistry micrographs of Beclin 1 protein expression in the hepatic tissue of animals (Red arrows indicate positive staining of target protein; images at 400× magnification; scale bar: 20 µm). (**B**) Beclin 1 protein quantification was shown in the bar chart as the area % (based on six non-overlapping microscopic fields). For *n* = 6, the values were shown as mean ± S.E.M. ** *p* < 0.01 vs. control; ^#^ *p* < 0.05 vs. DOX group. PTX, pentoxifylline; DOX, doxorubicin.

In order to determine whether autophagy was associated with the beneficial effects of PTX, immunohistochemistry was used to measure the protein expression of SQSTM-1/p62, which accumulates within cells in case of defective autophagy [12]. In the study, we also investigated the role of hepatic Beclin 1 levels in response to autophagy [26]. The present findings revealed that DOX significantly increased hepatic SQSTM-1/p62 protein expression (*p* < 0.0001) by 287.1% (Figure 7B), implying defective autophagy events. In tandem, DOX significantly reduced the hepatic protein expression of Beclin 1 (*p* < 0.01) by 65% (Figure 8B), corroborating the detection of defective autophagy. Following PTX administration to DOX-intoxicated animals, the defective autophagy events were improved, as denoted by the significant reduction in SQSTM-1/p62 (*p* < 0.001) by 46.5% alongside Beclin 1 significant upregulation (*p* < 0.05) by 108.3%. These data reveal that the observed autophagy enhancement by PTX is, at least partly, involved in its favorable outcomes against DOX-evoked hepatic pathological signs. 

### 2.6. Hepatic AMPK/mTOR Pathway Was Activated by PTX in DOX-Intoxicated Rats

**Figure 9:**
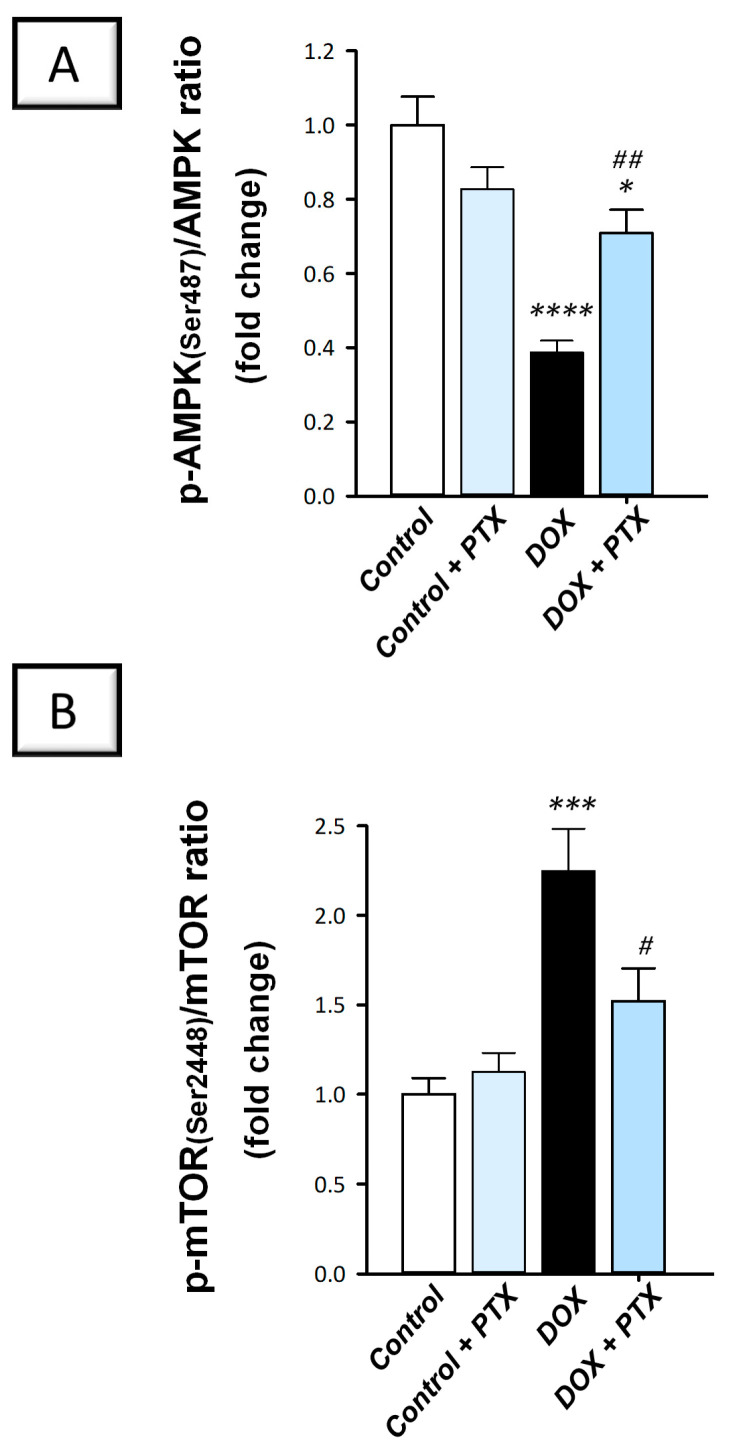
Pentoxifylline stimulated hepatic adenosine-monophosphate-activated protein kinase/mechanistic target of rapamycin (AMPK/mTOR) pathway in doxorubicin-intoxicated rats. Pentoxifylline (PTX; 100 mg/kg) was orally received daily, while doxorubicin (DOX; 18 mg/kg total cumulative dose; 3 mg/kg dose weekly) was received by the intraperitoneal route. The treatments were administered for 6 weeks. Herein, the protein expression of hepatic p-AMPK(Ser487), total AMPK, p-mTOR(Ser2448), and total mTOR was determined by ELISA. In the hepatic tissue of DOX-intoxicated rats, PTX resulted in an elevated p-AMPK(Ser487)/AMPK ratio (**A**) and diminished p-mTOR(Ser2448)/mTOR ratio (**B**). For *n* = 6, the values were shown as mean ± S.E.M. * *p* < 0.05, *** *p* < 0.001, or **** *p* < 0.0001 vs. control; ^#^ *p* < 0.05, *^##^ p* < 0.01 vs. DOX group. PTX, pentoxifylline; DOX, doxorubicin.

Evidence suggests that the AMPK/mTOR pathway positively impacts autophagy progression [12]. The present findings revealed that DOX significantly diminished hepatic p-AMPK/total AMPK (*p* < 0.0001) by 61.3% (Figure 9A), a known activator of autophagy response. In addition, DOX significantly elevated p-mTOR/total mTOR, a recognized autophagy inhibitory signal (*p* < 0.001) by 124.8% (Figure 9B), indicating AMPK/mTOR pathway suppression. Following PTX administration to DOX-intoxicated animals, the inhibition of the AMPK/mTOR pathway was counteracted as revealed by the significant reduction in p-mTOR/total mTOR (*p* < 0.05) by 32.3% and the significant increment in p-AMPK/total AMPK (*p* < 0.01) by 83.2%. These findings demonstrate that the observed AMPK/mTOR pathway stimulation by PTX is, at least partly, implicated in ridding hepatic cells of misfolded macromolecules and improvement in DOX-evoked pathological signs. 

## 3. Discussion

**Figure 10:**
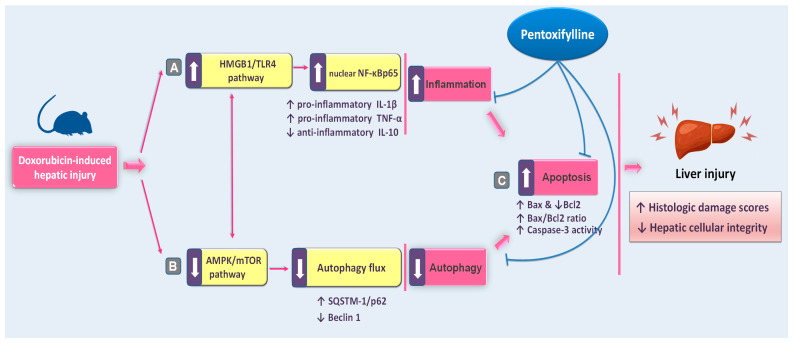
A concise overview of the proposed mechanisms for pentoxifylline’s mitigation against doxorubicin-evoked hepatic damage in rats. Based on the current findings, pentoxifylline attenuated the pathological signs of hepatic injury and improved serum hepatic markers. Mechanistically, these actions were elicited by (**A**) dampening hepatic pro-inflammatory events with the suppression of the HMGB1/TLR4/NF-κB axis; (**B**) rescuing the impeded autophagy events with activation of the AMPK/mTOR pathway; and (**C**) attenuation of the hepatic pro-apoptotic response, favoring hepatocyte survival and hepatic tissue preservation. Arrows: there are two types of arrows: those with a solid line denote activation and those with a blunt line denote inhibition.

In this study, we provide the first evidence suggesting that pentoxifylline (PTX) could be an effective approach to mitigating the hepatic damage triggered by doxorubicin (DOX) in rats. Herein, PTX administration to DOX-intoxicated rats attenuated the pathological signs of hepatic injury and improved serum hepatic markers revealing rescued hepatic cellular integrity. Moreover, we show that this mitigation was mainly elicited by curtailing the hepatic HMGB1/TLR4/NF-κB pro-inflammatory axis and pro-apoptotic responses alongside rescuing impaired autophagy with AMPK/mTOR pathway stimulation (Figure 10).

As an effective anticancer agent, DOX is applied in the management of various cancer types, such as solid tumors and hematologic malignancies. Yet, DOX’s long-term use can result in irreversible organ damage in vivo, including serious liver damage which limits DOX’s clinical application [3,4,6]. Indeed, hepatotoxicity is a frequent event following the administration of DOX to cancer patients and experimental animals [14]. Ample evidence exists that DOX treatment results in several structural and biochemical changes in rodents’ livers [3,6]. In perspective, diverse pathological signs of liver injury have been reported in animal models of DOX-induced hepatotoxicity, including central vein congestion, parenchymal necrosis, hepatocyte degeneration, edema, and leukocyte influx [3,4,27]. Moreover, DOX elevates serum levels of transaminases, including ALT and AST, which indicates deteriorated hepatocyte integrity [1,3,6]. Consistent with the previous literature, the current findings showed that DOX-intoxicated animals also exhibited similar degenerative histopathological aberrations and impaired hepatocyte integrity. As a result of these findings, our animal model has proven successful. Interestingly, PTX mitigated these changes, implying its competence in mitigating DOX-induced hepatotoxicity. These data also support the previously reported hepatoprotective actions of PTX in other liver injury models induced by hepatic ischemia/reperfusion [28], experimental autoimmune hepatitis [21], and fibrosis [29] alongside toxins such as carbon tetrachloride and acetaminophen [30]. 

Several studies have reported the implication of inflammation in the pathogenesis of DOX-induced hepatic injury [3,4,6]. As an activator of TLR4, the HMGB1 protein contributes to the production of pro-inflammatory mediators, which sustains an inflammatory environment [26,31]. In the same regard, several types of cells, including Kupffer cells, hepatocytes, sinusoidal endothelial cells, and hepatic stellate cells, have been reported to express TLR4 [31]. Binding of HMGB1 to its receptor TLR4 results in NF-κB activation that serves as a transcription factor for diverse pro-inflammatory chemokines and cytokines including IL-1β and TNF-α. These events prompt the influx of leukocytes into the inflamed liver, culminating in damage exacerbation [31,32]. HMGB1 is believed to mediate hepatic injury in rodent models of hepatic ischemic reperfusion and acetaminophen intoxication [31,33]. However, this is the first evidence that reports the involvement of the HMGB1/TLR4 axis in DOX-evoked hepatotoxicity. Interestingly, the present findings revealed that PTX effectively inhibited the pro-inflammatory HMGB1/TLR4/NF-κB axis in the hepatic tissues of DOX-intoxicated animals. This was associated with dampened hepatic IL-1β and TNF-α with upregulation of the anti-inflammatory IL-10. The latter observation is likely due to NF-κB inactivation seen by its downregulated protein levels in the nuclear compartment [3]. Our findings for suppressed inflammation in response to PTX align with previous studies that reported the anti-inflammatory and immunomodulatory effects of PTX in several experimental models [18,20,21,34]. In a rat model of rhabdomyolysis-induced acute renal damage, PTX improved the renal function markers and attenuated the pathological signs of kidney damage by dampening the TLR4/NF-κB pathway and the expression of IL-1β and TNF-α [20]. Likewise, PTX demonstrated favorable protection against corneal damage by combating the intensified pro-inflammatory response and lowering the expression of TNF-α, thereby promoting corneal epithelial repair [35]. In a mouse model of autoimmune hepatitis, PTX demonstrated favorable hepatoprotection by improving serum aminotransferases and minimizing the hepatic inflammatory infiltrates through its marked anti-inflammatory actions that were proven by downregulation of TNF-α and interferon-γ [21]. 

In autophagy, damaged cellular organelles and cytoplasmic proteins are engulfed and enveloped into vesicles known as autophagosomes. The fusion of these vesicles with the lysosomes ultimately results in the formation of autolysosomes where degradation of encapsulated contents takes place and the end products are recycled by the cell [9]. Various stress signals such as inflammatory responses, hypoxia, and ROS have been reported to drive the autophagy events likely by AMPK activation and mTOR inhibition [1,9]. In the context of DOX-induced organ damage, defective [1,12,13], as well as overactive [10,11] autophagy events, have been characterized. In this study, we detected—for the first time—impeded autophagy in DOX-induced hepatic damage seen by SQSTM-1/p62 accumulation and dampened AMPK/mTOR pathway. Consistent with our findings, dampened degradation of SQSTM-1/p62 protein has previously been identified as a marker for impaired autophagy, prompting enhanced pro-apoptotic response [9]. In agreement with our data, several studies have documented that DOX-induced cardiotoxicity is associated with a defective autophagy response [1,12,13]. When impaired autophagy prevails within cells, damaged proteins/organelles accumulate, allowing apoptotic cell death to occur with consequent tissue injury [1,9]. In tandem, evidence exists that the cleaved caspase-3 impairs autophagy by degrading the autophagy signal Beclin 1, thereby impairing autophagy initiation under pro-apoptotic conditions [1]. 

Interestingly, the present findings revealed the competence of PTX to rescue the impaired autophagy response and stimulate the AMPK/mTOR cascade. In line with our data, studies have indicated that stimulating autophagy can protect against DOX’s harmful effects. For example, rapamycin, an autophagy flux activator through mTOR inhibition, mitigates DOX-evoked cardiomyopathy in mice [1]. It has also been reported that resveratrol, in combination with moderate caloric restriction, can activate AMPK, resulting in ULK1 complex phosphorylation and autophagy initiation. These events trigger favorable cardiac functional outcomes [1,9]. At the molecular levels, autophagy stimulation is hypothesized to remove toxic protein aggregates and damaged mitochondria, favoring cell survival [1]. Hence, modalities that favor autophagy progression and autophagosome formation have been suggested to limit cell death and dampen tissue damage [36]. Indeed, evolving evidence has demonstrated favorable pro-autophagic features of PTX in experimental models. For instance, in an acute lung injury model, PTX effectively rescued the pulmonary injury by attenuating alveolar collapse and inflammatory infiltration by prompting pulmonary autophagy with upregulation of Beclin1 and LC3II/1 and dampening the apoptotic machinery in rats [18]. Regarding the observed AMPK/mTOR cascade activation by PTX, it has been reported that activation of this pathway stimulates the autophagy response by lowering mTORC1 levels and triggering autophagy machinery to initiate [9]. 

Apoptosis has been characterized to play a crucial role in the pathology of DOX-induced hepatic damage [27]. The induction of cell death in hepatocytes has been linked to the instigation of apoptotic machinery, including intrinsic mitochondrial signaling [27,28,29]. As a result of increased intrinsic apoptotic signals such as Bax, mitochondrial membrane permeabilization is enhanced with the consequent release of cytochrome c from the mitochondria into the cytoplasm. This event ultimately results in stimulation of the executioner caspase 3 activation and hepatocyte death [10,11,28,29]. These data coincide with the present findings that revealed upregulated apoptotic events, seen by intensified Bax levels and caspase 3 activity. In the current study, these deleterious responses were counteracted by PTX administration, as evidenced by Bax downregulation, Bcl2 upregulation, and suppressed caspase 3 activity, resulting in hepatocyte preservation and favorable survival. Indeed, the previous literature has characterized the marked anti-apoptotic features elicited by PTX in a rodent model of autoimmune hepatitis. In perspective, PTX guarded against hepatic damage by dampening the extrinsic apoptotic pathway that plays a crucial role in the pathology of autoimmune hepatitis by downregulating the expression of Fas, FasL, and TNFR1 [21]. 

HMGB1 has been characterized as a crucial signal for controlling the crosstalk among inflammation, autophagy, and apoptosis [14]. In this context, HMGB1 downregulation has been reported to correlate with suppressed apoptosis and enhanced autophagy in experimental models of cardiotoxicity [14], diabetes [15,37], and testicular damage [38]. In perspective, low levels of HMGB1 can contribute to the enhancement of autophagy under curtailed prooxidant conditions via activation of Beclin-1-dependent autophagy events [15,39]. These data align with the present study’s outcomes where PTX dampened HMGB1 overexpression, stimulated autophagy events, and inhibited apoptotic cell death. This is also corroborated by the observed Bcl2 upregulation which prevents Bax translocation into the mitochondria, thereby slowing the intrinsic pathway of apoptosis. Generally, the literature has demonstrated that modalities targeting HMGB1 downregulation have been demonstrated as successful tools for combating hepatic injury by raising the autophagy/apoptosis ratio [15]. Notably, Tang and co-workers [16] previously reported autophagy flux regulation by HMGB1 where HMGB1 controls autophagosome maturation and SQSTM-1/p62 degradation. 

Research on PTX’s role in cancer therapy revealed significant anti-cancer properties in human liver cancer cell line HepG2 cells by inhibiting their proliferation and inducing apoptosis. PTX also halted the growth of implanted HepG2 cells in mice, favoring apoptosis [40]. Meanwhile, PTX has been proven to dampen the migration of melanoma cells [41]. By inhibiting inflammation, one of the hallmarks of cancer, PTX exhibits beneficial anticancer features [19]. In perspective, PTX combats inflammation by suppressing the antigen-triggered activation of T and B lymphocytes [42]. Regarding the combination of PTX with DOX, studies have shown that PTX lowers DOX’s DNA binding and minimizes its side effects [19]. Importantly, ample evidence has revealed that PTX does not interfere with DOX’s cytotoxicity in several cancer cell lines and it can even synergistically enhance the cytotoxicity of DOX, thereby promoting its therapeutic ability. For instance, in human chronic myeloid leukemia cells, PTX synergistically augments DOX cytotoxicity by increasing DOX accumulation in these cancer cells, culminating in the amelioration of DOX’s resistance [43]. The combination of PTX and DOX has also demonstrated synergistic activity and dampened cellular proliferation in breast cancer cell lines [44]. Together, these data substantiate the notion that the co-administration of DOX and PTX does not compromise DOX’s ability to exert its cytotoxic impact on cancer cells and may even enhance DOX’s therapeutic efficacy. In the same context, evidence revealed that HMGB1 inhibitors play a significant role in limiting the chemoresistance of hepatocellular carcinoma (HCC) cells to DOX, resulting in the re-sensitization of DOX-treated HCC cells [45]. In light of the present findings, we demonstrated the efficacy of PTX in inhibiting the HMGB1 signal, an event that can be potentially useful for targeted treatment strategy in HCC.

## 4. Materials and Methods

### 4.1. Animals

The current work involved 24 adult male Wistar albino rats weighing 200 ± 20 g and aged 10 weeks. A one-week adaptation period was applied to animals. Experiments were conducted in housing conditions with a 12L/12D lighting cycle, regulated temperature (22–25 °C), and 40–50% humidity. All protocols included in this work were endorsed by the Institutional Research Ethics Committee (Ethical Reference No: NODCAR/I/28/2022 and 45-080). Moreover, animal handling and experimental methodologies were conducted in agreement with the Guide for Laboratory Animal Care and Use (Publication # 85-23, US-NIH).

### 4.2. Chemicals

The pharmaceutical company Ebewe Pharma (Unterach, Austria) provided doxorubicin hydrochloride whereas Hikma Pharmaceuticals (Cairo, Egypt) provided pentoxyphylline. Unless otherwise noted, Sigma-Aldrich provided all additional compounds and reagents (St. Louis, MO, USA).

### 4.3. Experimental Protocol

Rats were randomly distributed by a blinded technician into 4 experimental groups (*n* = 6 each) as follows. **(1)** The **control group** intraperitoneally received saline injection (single dose/week) for 6 weeks. Moreover, rats were orally administered 0.5% carboxymethylcellulose sodium solution (CMC) daily by gavage for 6 weeks. **(2)** The **control + PTX group** intraperitoneally received saline injection (single dose/week) for 6 weeks. Moreover, rats were orally administered pentoxifylline (100 mg/kg) suspended in CMC daily by gavage for 6 weeks. **(3)** The **DOX group** received doxorubicin in a total cumulative dose of 18 mg/kg. This was achieved by receiving a 3 mg/kg dose of doxorubicin hydrochloride in normal saline/week for 6 weeks by intraperitoneal route. Moreover, the same group orally received CMC daily by gavage for 6 weeks. **(4)** The **DOX + PTX group** intraperitoneally received a 3 mg/kg dose of doxorubicin hydrochloride in normal saline/week for 6 weeks (a total cumulative dose of 18 mg/kg). Moreover, rats were orally administered pentoxifylline (100 mg/kg) suspended in CMC daily by gavage for 6 weeks. 

Based on prior research, the dose of pentoxifylline (100 mg/kg) was chosen where it is demonstrated to effectively dampen inflammation and the pathological signs in rodent models of D-galactosamine-evoked hepatotoxicity [22], autoimmune hepatitis [21], carbon tetrachloride and acetaminophen-evoked hepatic damage [30], lung injury triggered by chlorine [18] or infrarenal aortic cross-clamping [34], and rhabdomyolysis-induced acute kidney injury [20]. Of note, studies have revealed the safety of this low dose of pentoxifylline in rats when compared with the reported LD_50_ of pentoxifylline in rats via the oral route (1170 mg/kg) [17,18]. Meanwhile, the DOX dosage and regimen were chosen in accordance with previous studies showing DOX’s ability to instigate evident hepatotoxicity in rodents [3,4,6,27,46].

### 4.4. Tissue and Blood Collection

At the end of the study period, all rats were anesthetized (thiopental; 30 mg/kg by i.p. injection). An intracardiac puncture was performed once anesthesia had been confirmed. Supernatants from blood samples were separated and stored at −80 °C after centrifugation at 1000× *g* for 10 min. Under anesthesia, the animals were euthanized by cervical dislocation. For biochemical analysis, the liver was immediately collected, cleansed, and stored at −80 °C. Formalin buffered at 10% was used to preserve part of the freshly isolated liver and the preserved specimens were forwarded to the pathology laboratory for histopathological and immunohistochemical analysis (3 random specimens per experimental group). For biochemical assays, homogenization of a second part of the liver tissue was applied in RIPA buffer provided with phosphatase/protease inhibitors. The supernatant was collected from the homogenate after centrifugation for 15 min at 10,000× *g* and stored at −80 °C.

### 4.5. Liver Function Tests and Hepatic Inflammatory Cytokines

Serum ALT and AST were determined using colorimetric kits purchased from Elabscience Biotechnology Incorporation (Houston, TX, USA; Cat. No. E-BC-K235-S and E-BC-K236-M, respectively) following the manufacturer’s instructions. The final color’s intensity was read at a 450 nm wavelength. Hepatic IL-10 and TNF-α were quantified by enzyme-linked immunosorbent assay (ELISA) kits procured from Cloud-Clone Corporation (Houston, TX, USA; Cat. No. SEA056Ra and SEA133Ra, respectively). The assay of IL-1β was determined by an ELISA kit procured from Elabscience Biotechnology Incorporation (Houston, TX, USA; Cat. No. E-EL-R0012). The final color’s intensity was read at a 450 nm wavelength. 

### 4.6. Histopathology

Preservation of the fresh liver tissue was implemented in 10% buffered formalin for 24 h. Fixation, dehydration, and clearing were applied to the hepatic specimens. Then, samples were processed in paraffin wax and cut into 5-micrometer sections. A staining protocol was applied using the Hematoxylin–Eosin (H–E) staining procedure [47]. Each slide was dehydrated with 95% and 100% ethanol, followed by xylene. Slides were mounted with a drop of mounting media and covered with a coverslip. An optical microscope was used to examine and capture all images (Model MX5200L, Meiji Techno Co., Chikumazawa, Japan). In the current histopathology protocol, we examined three specimens from each group and we photographed six non-overlapping fields. To limit bias, an expert pathologist blinded to the identity of treatment groups conducted the histopathological assessment.

Hepatic microscopical damage was quantitively examined based on the area percentage of inflicted damage. The pathological damage was examined in terms of the karyolitic nuclei, necrosis, fatty degeneration, and hepatocyte degeneration. A 0–4 grading system was employed as previously described [48]. In brief, a lack of lesions in the hepatic tissue was graded as 0, lesion presence in <10% area of the hepatic tissue was graded as 1, lesion presence in 10–40% area of the hepatic tissue was graded as 2, lesion presence in 40–60% area of the hepatic tissue was graded as 3, and lesion presence in >60% of hepatic tissue area was graded as 4. 

### 4.7. Hepatic HMGB1/TLR4/NF-κB Axis

Hepatic HMGB1 content was measured using an ELISA kit procured from AFG Bioscience (Northbrook, IL, USA; Cat. No. EK720661) as recommended by the vendor. The levels of hepatic TLR4 and nuclear NF-κBp65 were determined by ELISA kits purchased from Elabscience Biotechnology Incorporation (Houston, TX, USA; Cat. No. E-EL-R0990 and Cat. No. E-EL-R0674, respectively) following the manufacturer’s instructions. The final color’s intensity was read at a 450 nm wavelength. In order to determine the level of expression of the NF-κBp65 protein, the assay was conducted in the nuclear extract. Isolating total nuclear proteins was carried out according to the manufacturer’s instructions (Cayman Chemical, Ann Arbor, MA, USA) using an extraction kit specifically designed for the nuclear content (Cat. No. 10009277). 

### 4.8. Measurement of Apoptotic Events

The hepatic activity of caspase 3 was determined using a colorimetric assay procured from Sigma-Aldrich (St. Louis, MO, USA; Cat. No. CASP-3-C), as recommended by the vendor. The final color’s intensity was read at a 405 nm wavelength. As instructed by the manufacturer and previous studies [49], the data were expressed as a fold change. Moreover, the protein levels of hepatic Bcl2 and Bax were determined by MyBioSource Incorporation ELISA kits (San Diego, CA, USA; Cat. No. MBS2881713 and Cat. No. MBS2512405, respectively) following the manufacturer’s instructions. The final color’s intensity was read at 450 nm wavelength.

### 4.9. Immunohistochemistry

To minimize bias, an expert pathologist blinded to the identity of treatment groups conducted the processing of specimens in immunohistochemistry. Beclin 1 and SQSTM-1/p62 protein levels were detected by immunohistochemical staining in paraffin-embedded hepatic sections according to a routine immunohistochemistry protocol [50]. In brief, the 5-µm sections were deparaffinized, cleared, and processed for antigen retrieval using citrate buffer (50 mM, pH = 6.8). Inhibition of tissue endogenous peroxidase activity was achieved by using hydrogen peroxide solution (0.3%). The blockade of sections was implemented with the aid of 5% BSA. At 4 °C, the sections were probed overnight with primary antibodies against Beclin 1 or SQSTM-1/p62 (dilution 1:100 dilution, Abcam, Cambridge, MA, USA, Cat. No. ab62557 and Cat. No. ab91526, respectively). After washing with PBS, the sections were incubated with the secondary antibody. Counterstaining was applied using hematoxylin stain and brown staining was carried out using DAB chromogen. The images were captured under a light microscope (Model MX5200L, Meiji Techno Co., Chikumazawa, Japan). In each experimental group, we imaged six non-overlapping fields. Fiji ImageJ^®^ software (Version 1.51r) was used to quantify the area of brown staining (NIH, Bethesda, MD, USA). 

### 4.10. Determination of the AMPK/mTOR Pathway

The hepatic content of p-AMPK and total AMPK was measured using a RayBiotech-specific ELISA kit (Norcross, GA, USA; Cat. No. PEL-AMPKA-S487-T), as recommended by the vendor. In this regard, the p-AMPK(Ser487) antibody was precoated in half of the wells to detect the phosphorylated form, whereas the pan AMPK antibody was precoated in the other half of the wells to detect the total form of AMPK. The final color’s intensity was determined at a 450 nm wavelength. Moreover, hepatic p-mTOR(Ser2448) and total mTOR levels were determined by ELISA kits supplied by Cell Signaling Technology, Danvers, MA, USA, Cat. No. 7976C and Cat. No. 7974C, respectively). As instructed by the manufacturer, the results were expressed as the ratio of phosphorylated/total form of each target protein. The final color’s intensity was read at 450 nm wavelength. 

### 4.11. Statistics

GraphPad Prism (version 8.0) was employed to run statistical analysis. Initially, the normal distribution of results was investigated with the Shapiro–Wilk test. For parametric (normally distributed) values, we checked the statistical significance among groups with the aid of one-way ANOVA. When significance was detected, we employed Bonferroni’s test for running multi-comparisons (at *p* < 0.05). For non-parametric values, we checked the statistical significance among groups with the aid of Kruskal–Wallis analysis. When significance was detected, we employed Dunn’s test for running multi-comparisons (at *p* < 0.05). 

## 5. Conclusions

The current findings provide evidence demonstrating the competence of PTX to dampen hepatic injury triggered by DOX in vivo in rats. Several molecular mechanisms were implicated in PTX’s actions, including suppressing the HMGB1/TLR4/NF-κB pro-inflammatory axis and pro-apoptotic events alongside rescuing impaired autophagy with AMPK/mTOR stimulation. Hence, the use of PTX for the management of DOX-evoked hepatic injury may be considered an adjunct modality. Yet, further studies are warranted to examine the efficacy of multiple doses of PTX in mitigating DOX-induced hepatic injury in vivo. Moreover, supplemental research is required to explore the efficacy of the PTX and DOX combination in inhibiting HepG2 cell proliferation in vitro. Clinical investigation is also suggested to examine PTX’s potential to limit DOX hepatotoxicity. 

## Data Availability

Data are contained within the article.
